# Blood autophagy defect causes accelerated non-hematopoietic organ aging

**DOI:** 10.18632/aging.102086

**Published:** 2019-07-21

**Authors:** Yixuan Fang, Lingjiang Zhu, Ni An, Gaoyue Jiang, Jiawei Qian, Ruijin Zhao, Na Yuan, Suping Zhang, Jianrong Wang

**Affiliations:** 1 Hematology Center of Cyrus Tang Medical Institute, Jiangsu Institute of Hematology, Institute of Blood and Marrow Transplantation, Collaborative Innovation Center of Hematology, Key Laboratory of Stem Cells and Biomedical Materials of Jiangsu Province and Chinese Ministry of Science and Technology, State Key Laboratory of Radiation Medicine and Radioprotection, Soochow University School of Medicine, Suzhou 215123, China; 2 Research Center for Non-medical Healthcare of Soochow University and Beijing Yaozhongtang, Cyrus Tang Medical Institute, Soochow University School of Medicine, Suzhou 215123, China

**Keywords:** autophagy, aging, blood, non-hematopoietic organ

## Abstract

Autophagy has been well studied in regulating aging; however, the impact of autophagy in one organ on the aging of other organs has not been documented. In this study, we used a mouse model with deletion of an autophagy-essential gene Atg7 in hematopoietic system to evaluate the intrinsic role of hematopoietic autophagy on the aging of non-hematopoietic organs. We found that autophagy defect in hematopoietic system causes growth retardation and shortened lifespan, along with aging-like phenotypes including hypertrophic heart, lung and spleen, but atrophic thymus and reduced bone mineral density at organismal level. Hematopoietic autophagy defect also causes increased oxidative stress and mitochondrial mass or aging gene expression at cellular level in multiple non-hematopoietic organs. The organ aging in the Atg7-deleted mice was reversed by anatomic connection to wild-type mice with intact blood autophagy via parabiosis, but not by injection of blood cell-free plasma. Our finding thus highlights an essential role of hematopoietic autophagy for decelerating aging in non-hematopoietic organs.

## INTRODUCTION

Organ function declines with age and the decline is attributed to progressive reduction of tissue stem cells or deterioration of their self-renewal and differentiation power [1–4], or reduction of certain blood components or systemic factors [5–9] that may support tissue stem cell microenvironment and tissue repair and regeneration. Historically, transfusion of blood or of its components has been attempted for rejuvenation of organ and whole body since a century ago [10]. Parabiosis, an anatomic procedure of joining two animals between the aged and young so that they share a combined blood circulation, has been used to study anti-aging effect by a younger partner, in particular the young blood [11–13].

Autophagy is a conserved process that catabolizes and removes unwanted intracellular components including macromolecules and organelles to maintain energy homeostasis and protect cells against stress. Autophagy is essential for maintaining a life-long hematopoiesis [14–16]. Autophagy has fairly recently been considered as an anti-aging mechanism, mediating many lifespan-extending and antisenescence cascades [17–21]. Enhanced autophagy has been shown to promote longevity, as activation of autophagy by either tissue-specific overexpression of single autophagy genes or other positive intervention on autophagy is sufficient to extend lifespan [18, 22, 23–27], whereas impaired autophagic capacity, by either deletion of autophagy-essential gene or blockade of autophagic flux, reduces lifespan and precipitates premature aging in numerous model species [28–32]. Autophagy-reporter gene analyses and autophagy gene expression studies in different species indicate a progressive decrease in autophagy activity over time with age. Furthermore, experiments used multiple model organisms to modulate autophagy gene activity indicate that autophagy activation can be used as a strategy to promote longevity [33–35]. It has been established that disruption of autophagy in hematopoietic system leads to blood aging [14, 19]. However, the impact of blood, in particular hematopoietic autophagy, on the aging of non-hematopoietic organs in the same organism has not been examined. In this study, we used a mouse model with deletion of an autophagy-essential gene Atg7 in hematopoietic system to provide evidence that hematopoietic autophagy is important in the opposition to the aging of non-hematopoietic organs.

## RESULTS

### Autophagy defect in hematopoietic system results in growth retardation and shortened lifespan

**Figure 1:**
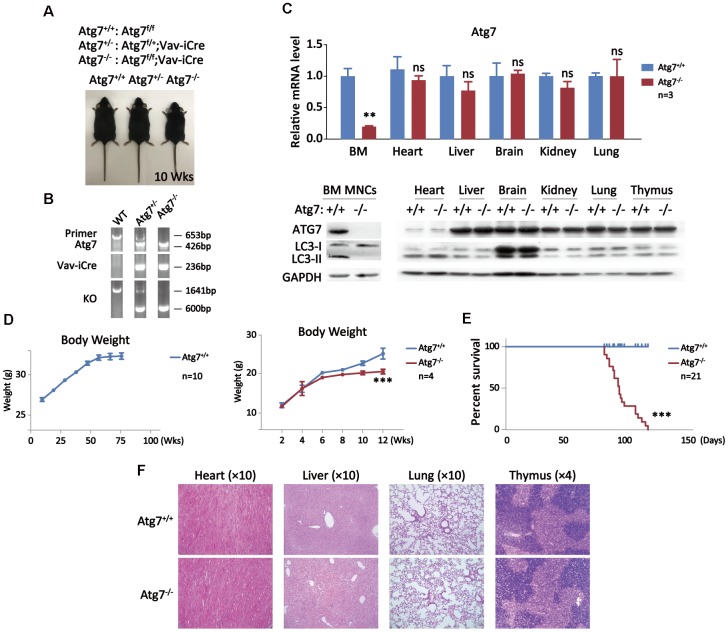
**Growth retardation and shortened lifespan of the mice with deletion of an autophagy-essential gene Atg7 in hematopoietic system.** (**A**) Three genotypes for wild-type, heterozygote, and homozygote for Atg 7 deletion in hematopoietic system with representative images of the mice. The images were taken at age of 10 weeks. (**B**) PCR Genotyping analysis of the offsprings from Atg7^f/f^ mice crossing Vav-iCre mice to screen Atg7^f/f^;Vav-iCre mice. The sequences for the primers used in PCR are given in the method section, and their PCR amplified bands representing specific genotypes were indicated in the agarose gel electrophoresis films. (**C**) Examination of Atg7 expression in wild-type and the Atg7-deleted mice. Upper panel, quantitative PCR analysis of Atg7 transcription normalized to Gapdh transcript in different organs; lower panel, western blotting analysis of autophagy-essential protein ATG7 and lipidation of LC3 in different organs. GAPDH used as a loading control. (**D**) Growth comparison between wild-type and Atg7-deleted mice. Wild-type mice progressively gain weight before age of 60 weeks (left panel), but Atg7-deleted mice cease weight gain at about age of 6 weeks (right panel). (**E**) Measurement of lifespan of wild-type and Atg7-deleted mice. (**F**) Immunohistological examination of heart, liver, lung and thymus from 10 weeks old wild-type and Atg7-deleted mice by HE staining.

To explore the role of hematopoietic autophagy on the impact on mammalian organismal aging, the autophagy gene Atg7, essential for two autophagic conjugation systems responsible for the formation of autophagosomes, was deleted by gene targeting in hematopoietic system of Atg7^f/f^;Vav-iCre mice [14–16]. Shown are representative images and PCR results of wild-type, monoallelic deletion of Atg7 (Atg7^f/+^;Vav-iCre) and biallelic deletion of Atg7 (Atg7^f/f^;Vav-iCre) mice by genotyping analysis, confirming that Atg7 gene is deleted in the Atg7^f/f^;Vav-iCre mice, designated as Atg7^-/-^ (Figure 1A, 1B). For analysis of non-hematopoietic tissues/organs, perfusion was performed and blood cells (Ter119^+^ or CD45^+^) were further removed by fluorescence-activated cell sorting. Quantitative RT-PCR result from the Atg7^f/f^;Vav-iCre mice shows that transcription of Atg7 was primarily reduced in the bone marrow, sparing other organs tested, including heart, liver, brain, kidney and lung in which blood cells were removed (Figure 1C, upper panel). Further examination with western blotting confirmed the absence of ATG7 protein solely in the bone marrow mononuclear cells, which are largely bone marrow hematopoietic cells (Figure 1C, lower panel). Clearly, the lipidation of LC3-I to LC3-II, a key step in autophagy, was disrupted since LC3-II was efficiently diminished in blood cells (Figure 1C, lower left panel), whereas LC3-I was processed to LC3-II by lipidation in the non-hematopoietic organs including heart, liver, brain, kidney, lung and thymus (Figure 1C, lower right panel) [36]. These genotypic and phenotypic results together prove successful autophagy blockade in the hematopoietic system, but not the non-hematopoietic organs with blood cells experimentally removed in the Atg7-deleted mice. Wild-type mice grew with an progressive increase in weight until around age of 60 weeks (Figure 1D, left panel), whereas mice with Atg7 deletion in the hematopoietic system displayed a growth retardation starting at early age of 6 weeks (Figure 1D, right panel) and had a significantly shortened lifespan of 120 days at maximum (Figure 1E), without detectable symptoms of a known disease or infection from immunohistological examination of the non-hematopoietic organs by Hematoxylin and Eosin (HE) staining (Figure 1F). Since Atg7 is solely implicated in autophagy, the above data indicate that autophagy intact in the hematopoietic system is essential for maintaining of a normal growth and lifespan.

### Blood autophagy defect leads to hypertrophy in several solid organs and reduction in bone mineral density

**Figure 2:**
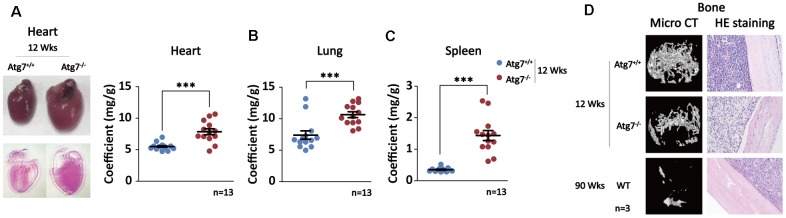
**Multiple aging-like organ abnormalities in the Atg7-deleted mice in hematopoietic system.** (**A**) Alteration of heart in morphology and size in the Atg7-deleted mice. Left panel, representative image of heart unstained (upper) and HE stained (down); right panel, heart/body weight ratio (coefficients) of the Atg7-deleted and wild-type mice. (**B**) Lung coefficients of the Atg7-deleted and wild-type mice. (**C**) Spleen coefficients of the Atg7-deleted and wild-type mice. (**D**) Alteration of bone mineral density. Upper, micro-CT analysis of bones of in the Atg7-deleted mice and the same-age wild-type mice as well as old wild-type mice; down, HE staining of bones from 12-week wild-type, Atg7-deleted, and old wild-type mice.

Given that blood autophagy defect causes significantly shortened lifespan without obvious signs of disease evidenced in the non-blood organs, we hypothesized that autophagy defect of hematopoietic system may promote non-hematopoietic organ aging that leads to shorter life. To this end, we examined the alteration of the non-hematopoietic organs from the mice with autophagy defect in hematopoietic system. The anatomic results with or without HE staining show significant cardiac hypertrophy in the Atg7-deleted mice at age of 12 weeks, shown by representative images and coefficients of organ (mg/g) (Figure 2A); similar hypertrophy in other organs including lung and spleen was also apparent based on their elevated coefficients of organ (Figure 2B), mimicking the previous report that organ aging is characterized by the presence of hypertrophy [37]. Micro-CT analysis reveals reduced bone mineral density in the Atg7-deleted mice as compared with the same-age wild-type mice, albeit the wild-type old mice at age of 90 weeks display much lower bone mineral density (Figure 2D, upper panel). Immunohistological assay with HE staining further confirmed a bone loss in the mice with autophagy defect in hematopoietic system, and the bone loss of the Atg7-deleted mice at age of 12 weeks is closer to that of the wild-type old mice at age of 90 weeks than that of the wild-type mice at age of 12 weeks (Figure 2D, lower panel). Therefore, autophagy defect in hematopoietic system leads to aging-like organismal abnormality in several non-hematopoietic organs.

### Blood autophagy defect gives rise to thymic atrophy

**Figure 3:**
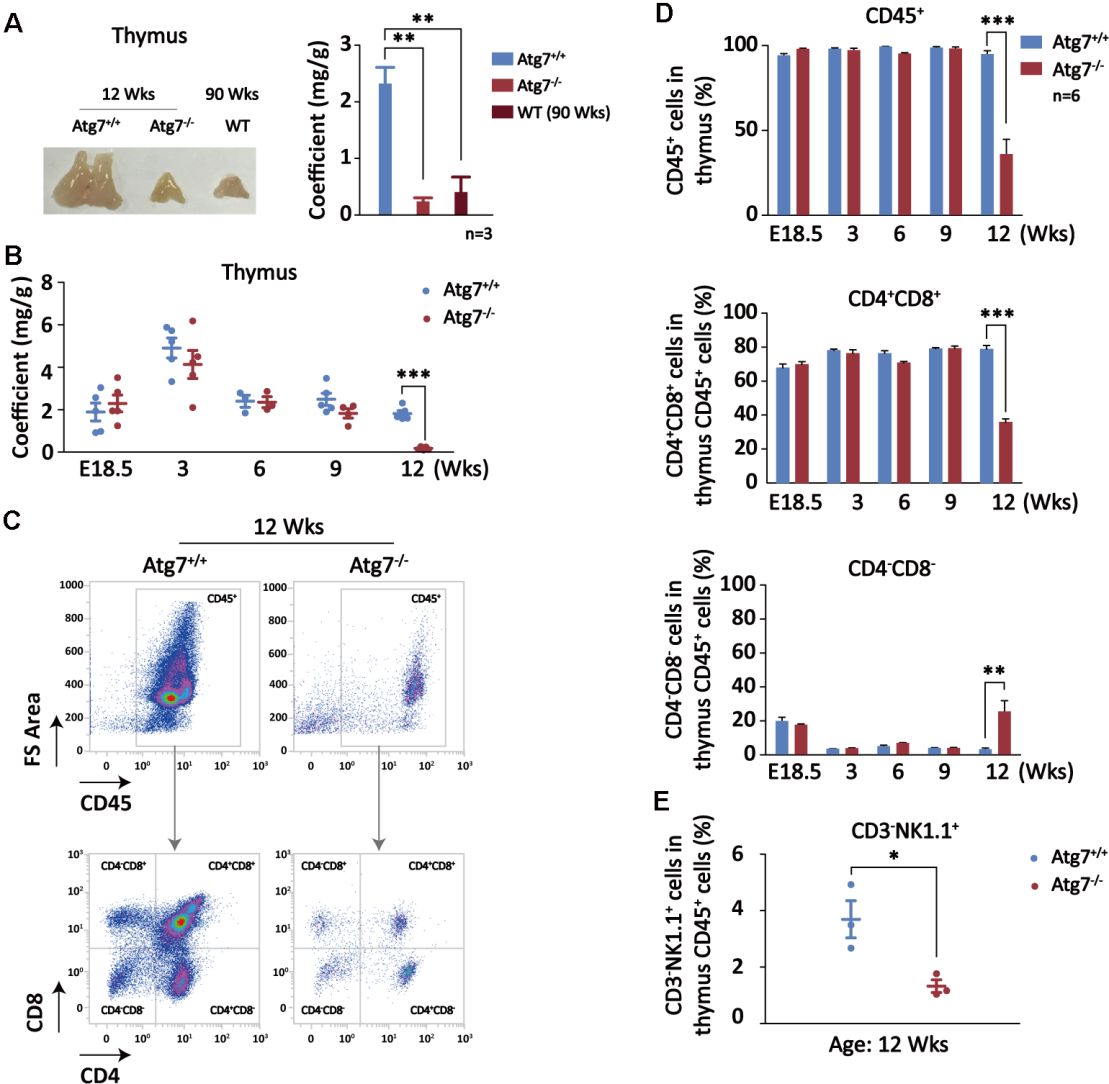
**Autophagy defect causes synchronous thymic atrophy and T cell (CD4^+^CD8^+^) reduction after mouse development is completed.** (**A**) Alteration of thymus in size and weight. Left, a representative picture of thymus; right, thymus coefficient of Atg7-deleted mice as compared with the same-age wild-type mice and the old wild-type mice. (**B**) Measurement of thymus coefficients in time points indicated in the entire lifespan of the Atg7-deleted mice (organ/body, mg/g). (**C**) Scheme for analysis of T cells by flow cytometry. Shown are representative flow images for quantification of total blood cell (CD45^+^) and T cells (CD4^+^,CD8^+^) in total thymus cells. (**D**) Statistical analysis of the percentages of T cell populations in total thymus blood cells in the entire lifespan of the Atg7-deleted mice. (**E**) Statistical analysis of the percentages of NK cell populations in total thymus blood cells in the Atg7-deleted mice at age of 12 weeks.

Although hypertrophy is often a reflection of aging for most of organs, reduced thymus is responsible for impaired maturation of T cells and thus is a major cause of organismal aging [38–40]. Indeed, the thymus was significantly reduced in the mice at age of 12 weeks with autophagy defect in hematopoietic system, and the size is surprisingly close to that of wild-type old mice at age of 90 weeks (Figure 3A). In order to explore if thymic atrophy is a consequence of aging caused by blood autophagy disruption, or a secondary cause that promotes non-hematopoietic organ aging, we examined the size and weight of thymus from embryonic E18.5 to postnatal age of 12 week when the mice with disrupted blood autophagy start to succumb. After birth, thymus reaches its largest relative size around age of 3 weeks. Although autophagy is constitutively disrupted in the hematopoietic system, thymus in size and weight was not altered in embryonic development and young age until age of 12 weeks when thymic atrophy was significant, manifested by co-efficient analysis (Figure 3B). Similarly, hypertrophy in non-hematopoietic organs became apparent at the age when thymic atrophy occurs in the Atg7-deleted mice (data not shown). These observations suggest that thymic atrophy is not an immediate consequence from blood autophagy disability, nor a secondary cause responsible for non-hematopoietic organ aging; rather, it appears to be an accumulated aging consequence pertinent to autophagy disruption.

T cell development and maturation is the core cascade in the thymus and T cells is one of major forces fighting against aging [41]. Blood cells and T cells in percentage in the thymus were analyzed with flow cytometry (Figure 3C). At age of 9 weeks, reduction in thymus blood cells (CD45^+^) and T cells in thymus blood cells were not found, but at age of 12 weeks, total blood cells in thymus was decreased by about 70% (Figure 3D, upper panel), whereas CD4, CD8, and CD4CD8 cells were all significantly reduced (Figure 3C). In particular, percentage of CD4^+^CD8^+^, the major T cell population, in the thymus CD45^+^ cells was reduced at least by half (Figure 3D, left lower panel). Despite of an increase in percentage, CD4^-^CD8^-^ population that accounts for a minor portion in thymus blood cells was also decreased (Figure 3C). These results suggest that hematopoietic autophagy defect affected T cells primarily on CD4 and CD8 cell populations in the thymus.

Physiologically, a small portion of natural killer (NK) cells differentiate and mature in thymus before they enter into the circulation. NK cells have recently been found to be implicated in the regulation of aging [42, 43]. To explore the effect on thymus NK cells in the blood autophagy defective mice, we examined the percentage of NK cell population using CD3^-^NK1.1^+^ markers in the total thymus blood cells (CD45^+^) in flow cytometry. The result shows that NK cell population in percentage was significantly reduced (Figure 3E), similar to the pattern of T cells at mouse age of 12 weeks. This reduction may also be more or less contributed to the mouse aging.

### Hematopoietic autophagy defective mice display cellular aging phenotypes in multiple non-hematopoietic organs

**Figure 4:**
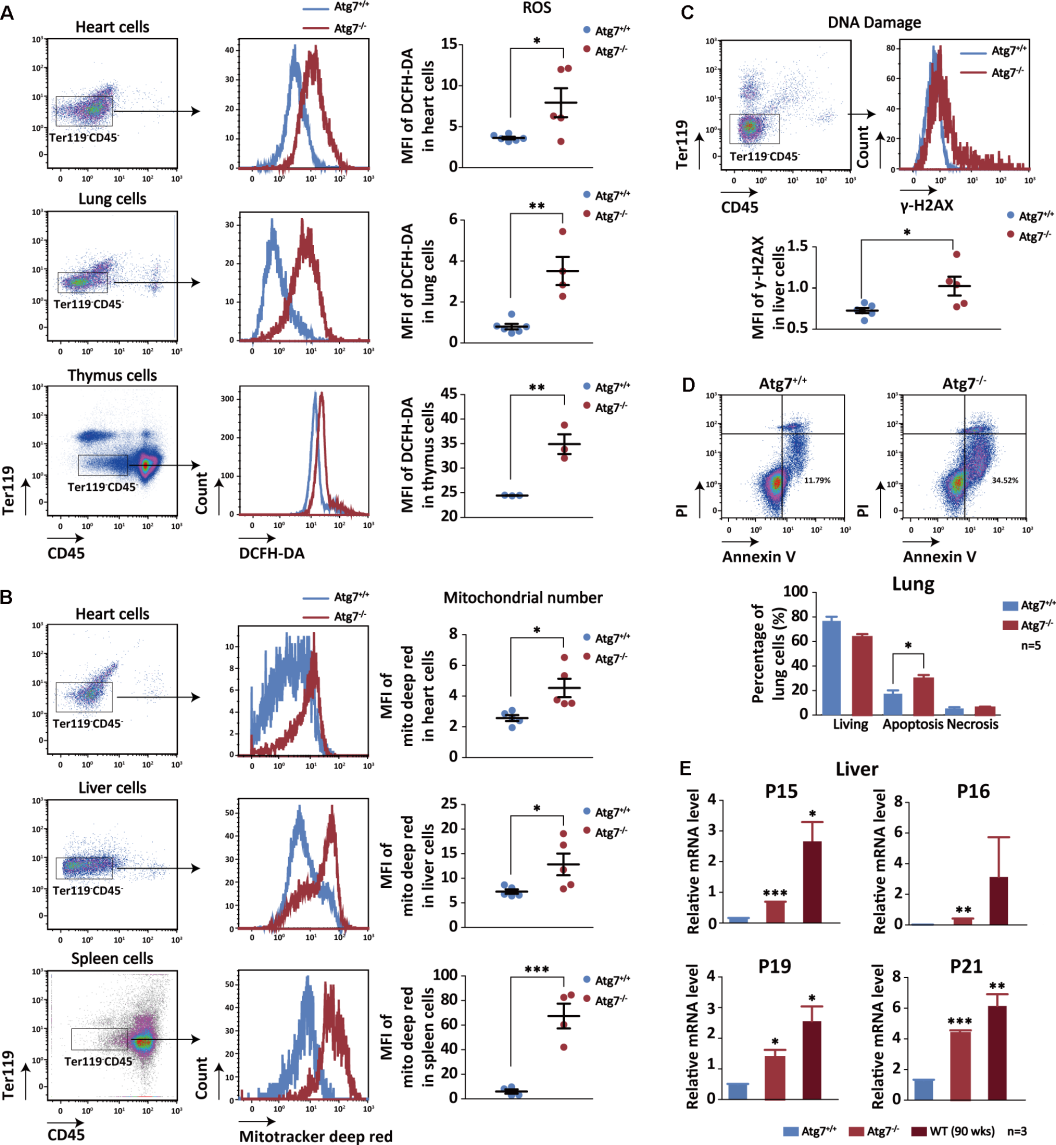
**Increased cellular aging markers in the non-hematopoietic organs of the mice with hematopoietic autophagy defect.** (**A**) Flow cytometric analysis of ROS levels of the heart, lung and thymus cells with fluorescein DCFH-DA. Left and middle, gating strategy for the flow-cytometric assessment of non-hematopoietic cells (CD45^-^Ter119^-^); right, geometric mean fluorescence intensity (MFI) of DCFH-DA in the heart cells of wild-type mice and Atg7-deleted mice. (**B**) Flow cytometric analysis of mitochondrial mass levels of the heart, liver and spleen cells with florescent Mitotracker Deep Red. Left and middle, gating strategy for the flow-cytometric assessment of non-hematopoietic cells (CD45^-^Ter119^-^); right, geometric mean fluorescence intensity (MFI) of MitoTracker Deep Red in the heart or liver cells of wild-type and Atg7-deleted mice. (**C**) Flow cytometric analysis of DNA damage with γ-H2AX. Upper, gating strategy for non-hematopoietic cells (CD45^-^Ter119^-^) in the liver; lower, geometric mean fluorescence intensity (MFI) of γ-H2AX in the liver cells. (**D**) Analysis of apoptosis and necrosis in the lung cells of wild-type mice and Atg7-deleted mice by annexinV and PI double staining. Upper, representative flow cytometric measurement; lower, statistical results from cytometric analysis. (**E**) Quantitative RT-PCR analysis of four aging related genes (P15, P16, P19, P21) in the liver cells (blood cells removed by sorting against CD45^+^ or Ter119^+^) of young wild-type mice, Atg7-deleted mice and old wild-type mice.

To further test our hypothesis that blood autophagy may be indispensible for regulating non-hematopoietic organ aging, we set to measure cellular aging cascades in the non-hematopoietic organs of the mice with autophagy defect in hematopoietic system. The non-hematopoietic cells from the solid organs were sorted for aging analysis with antibodies for CD45^-^Ter119^-^ populations by flow cytometry to exclude blood cells. Oxidative stress is a universal hallmark of cellular aging [44, 45]. Flow cytometric analysis with DCFH-DA shows increased reactive oxygen species (ROS) level in the non-hematopoietic cells of the heart, lung and thymus in the Atg7-deleted mice (Figure 4A). Mitochondrial number, which is alternatively named mitochondrial mass and measured by MitoTracker Deep Red reagent in flow cytometer, is also a reflection of metabolic and oxidative stress level of the cells of interest [46–48]. The results show that mitochondrial mass was increased in the heart, liver and spleen cells of the Atg7-deleted mice (Figure 4B). γ-H2AX is a reliable indicator for DNA damage response that is a strong indicator for aging [49]. Flow cytometric analysis with antibody against γ-H2AX shows that DNA damage level was elevated in the liver cells of the Atg7-deleted mice (Figure 4C). Increased apoptosis is associated with organismal aging process. Flow cytometric analysis on lung cells by annexinV and PI double staining suggests that the Atg7-deleted mice displayed an increase in apoptosis but not necrosis in the lung cells (Figure 4D). Finally, detection of four aging genes including P15, P16, P19, and P21 in the liver cells reveals that all of the expression levels for these four genes were increased in the Atg7-deleted mice at age of 12 weeks, reaching closer to those of the old mice at age of 90 weeks (Figure 4E). Therefore, hematopoietic autophagy defect results in cellular aging phenotypes in the non-hematopoietic organs.

### Mice with intact blood autophagy reversed the organ aging in the Atg7-deleted mice via parabiosis

**Figure 5:**
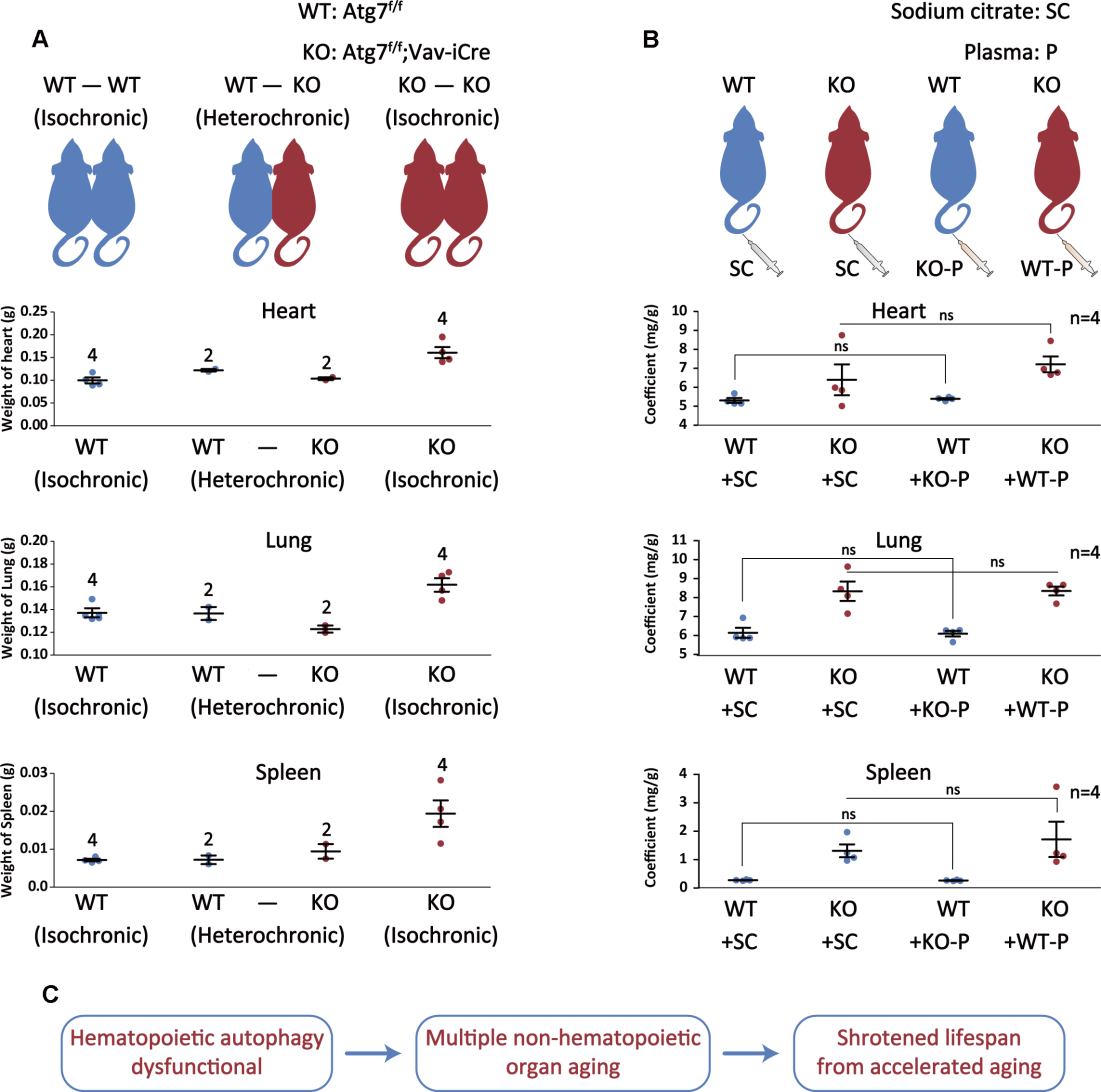
**Autophagy-intact blood cells not plasma reverse autophagy defect-caused aging of non-hematopoietic organs.** (**A**) Upper, schematic depicting the parabiotic pairings; lower, graph representing the heart/lung/spleen weight after 4 weeks of parabiosis. (**B**) Upper, schematic illustrating plasma treatment; lower, graph representing the heart or lung or spleen/body weight ratio after plasma treatment. (**C**) Summary of the study. Autophagy defect in hematopoietic system accelerates multiple non-hematopoietic organ aging, ultimately leading to a much shortened lifespan of the mouse.

To confirm blood autophagy is important for slowing the aging of non-hematopoietic organs, we used parabiosis [11] to examine the contribution to mitigation of organ aging of Atg7-deleted mice by autophagy-intact blood. The results show that the weight of heart, lung and spleen was obviously normalized by about 30 to 60% in organ co-efficient in the Atg7-deleted mice by anatomical connection to wild-type mice with intact autophagy, as compared with the positive control (WT-WT) and negative control (KO-KO) (Figure 5A). Shared blood circulation by two mice contains not only blood cells, but also plasma. To determine whether the rejuvenation of organs was contributed by blood cells or plasma, or by both, we injected plasma into the Atg7-deleted mice and wild-type mice. The results show that injection of plasma from wild-type mice with intact autophagy did not improve the normalization of the organ weight for the Atg7-deleted mice (Figure 5B). Therefore, it appears that blood cells, not plasma, from wild-type mice, reversed the organ aging in the autophagy-defective mice in the parabiosis experiment.

## DISCUSSION

The present study aimed to examine the impact of hematopoietic autophagy on the aging of non-hematopoietic organs. We used a conditional gene knockout mouse model with Atg7 deletion in hematopoietic system to assess the consequences on the non-hematopoietic organs. We found that autophagy defect in hematopoietic system causes growth retardation and shortened lifespan with hypertrophy in heart, liver and spleen, but atrophy in thymus; hematopoietic autophagy defect also reduces bone mineral density, increases oxidative stress and aging gene expression in multiple non-hematopoietic organs, and this defect in aging can be reversed by input of blood cells with intact-autophagy, but not plasma.

The mechanisms underlying organismal aging are not fully understood; however, oxidative stress has been identified to contribute to aging in both model animals [50–52] and humans [53, 54], and this may increase the risk of diseases such as stroke, and coronary heart disease. Mechanistically, the age-related increase in reactive oxygen species may partially be attributed to the increased activity of nicotinamide adenine dinucleotide phosphate-oxidase in vessel walls [50, 53, 55, 56]. In this study, we found that autophagy defect in hematopoietic system causes aging-like organ abnormality, increased levels in oxidative stress and mitochondrial mass in multiple non-hematopoietic organs. Our observation is thus in consistency with previous report that organismal aging is reflected by the presence of aging-like organs and accumulation of dysfunctional mitochondria [37].

Thymus is a center of combating organismal aging via output of matured T cells [57]. While genetic manipulation of T cell production that causes thymic atrophy may not be necessarily related to aging, atrophy of the thymus is often associated aging in immune system [58]. Age-associated thymic atrophy results in diminished generation of T cells. Age-associated thymic atrophy occurs in the aged population, leading to gradual decline in the ability of the immune system to respond to various antigens. Notably, one of the most significant hallmarks of human immune aging is the decrease in the absolute number and percentage of peripheral blood CD8 T-cells [59], which could be often attributed to thymic atrophy [40]. In addition, thymic atrophy is associated with various stress conditions such as infections and oncogenesis [60]. Although numerous factors have been implicated in studies to be responsible for age-related thymic atrophy and a variety of stimuli are known to regulate transient thymic atrophy, mechanisms regulating progressive age-associated atrophy have been difficult to resolve [60, 61]. This is largely due to the diversity of periodic, episodic, and cumulative events that influence thymus size and function over the lifespan [61]. It is also due to the fact that one of the primary targets of age-associated thymic atrophy is a relatively rare population, thymic stromal cells [61].

To determine if thymic atrophy caused by autophagy defect in hematopoietic system is possibly associated with mouse development or aging, we examined the thymus size and T cell numbers in the thymus during and after mouse development in the Atg7-deleted mouse model. Although autophagy is constitutively disrupted in hematopoietic system, thymus in size and weight was not altered in embryonic development and in early postnatal age. Thymus begun to reduce in size at age of 9 weeks or later. In contrast, at age of 9 weeks, neither blood cells in thymus cells nor CD4^+^CD8^+^ T cells in thymus CD45 cells was altered in the Atg7-deleted mice. It appeared that thymic atrophy may occur earlier, at least not later, than reduction in T cells. At age of 12 weeks, the thymus coefficient and T cells were significantly reduced. In our previous study, apoptosis or necrosis of lymphocytes was not significantly found in the Atg7-deleted mice [16]. Therefore, reduction in T cells may be attributed to less output, not more death in the thymus. Nevertheless, in the present study, it is difficult to determine whether thymic atrophy causes T cell reduction, or vise versa. Likewise, it also lacks in evidence that thymic atrophy and T cell reduction contribute to non-hematopoietic organ aging in the context of blood autophagy abnormality. In particular, the reduction of T cells was almost synchronized with the thymus atrophy, thus precluding our interpretation, with current data available, on the possible mechanism by which thymus and T cells function in response to autophagy defect-triggered aging of non-hematopoietic organs. However, it is has been known that disrupted autophagy in hematopoietic system causes blood aging [14, 19]. Our present study showed that autophagy defect shortened lifespan of the mice without an apparent phenotype of diseases or infection, and several aging-like markers were upregulated in multiple non-hematopoietic organs. Furthermore, aging phenotypes in the autophagy-defective mice could be reversed by parabiosis with autophagy-intact mice, but not by injection of plasma. Therefore, in contrast to previous reports on the role of autophagy in aging of the local tissue or organ, our data indicate that loss of blood autophagy leads to accelerated aging of the non-hematopoietic organs, and autophagy of hematopoietic system decelerates aging in non-hematopoietic organs. Since blood connects to all organs in the body by providing oxygen and nutrients, it is very likely that blood aging is one of the major causes responsible for acceleration of non-hematopoietic organ aging and reduction of lifespan (Figure 5C). Therefore, future work is necessary to determine how hematopoietic autophagy defect by constitutive deletion of an autophagy-essential gene mechanistically compromises aging agenda in the non-hematopoietic organs.

## MATERIALS AND METHODS

### Animals and genotyping

The generation of genetically modified mice Atg7^f/f^;Vav-iCre has been previously described [15, 62]. All mice were bred and housed in the specific pathogen free animal facilities of Soochow University. For genotypic analysis, DNA was extracted by Genomic DNA Mini Preparation Kit with Spin Column (Beyotime Biotechnology, D0063), and floxed p sites were amplified by specific primers (F- CATCTTGTAGCACCTGCTGACCTGG, R1-CCACTGGCCCATCAGTGAGCATG, R2-GCGGATCCTCGTATAATGTATGCTATACGAAGTTAT). Sequence of iCre was detected by primers (F-AGATGCCAGGACATCAGGAACCTG, R-ATCAGCCACACCAGACACAGAGATC). Knockout of Atg7 was detected by primers (F-TGGCTGCTACTTCTGCAATGATGT, R-AAGCCAAAGGAAACCAAGGGAGTG). DNA bands were detected by agarose gel electrophoresis and analyzed by Gel DocXR. Atg7^f/f^ serves as the control mouse Atg7^+/+^ in this study. All experimental procedures with animals were approved by Soochow University Institutional Animal Care and Use Committee.

### Western blot analysis

Cell lysates were obtained by homogenizing organs in lysis buffer, followed by centrifugation. Proteins were separated by polyacrylamide gels electrophoresis, and transferred to hydrophobic polyvinylidenedifluoride membranes (EMD Millipore, IPVH00010). Nonspecific binding was blocked by 5% skim milk. Membranes were probed with primary antibodies to ATG7 (Abcam, ab133528), LC3 (Novus, NB100-2220), or GAPDH (Proteintech, 60004-1), and then treated with secondary antibodies. Protein bands were detected by chemiluminescence detection kit for HRP (Biological Industries, 20500120).

### RNA isolation and quantitative RT-PCR analysis

Total RNA was extracted by trizol (Ambion, 15596018). Subsequently, total RNA was reverse transcribed to cDNA using the RevertAid First Strand cDNA Synthesis Kit (Thermo Fisher Scientific, K1622) following the manufacturer’s instructions. Quantitative real-time (RT) polymerase chain reaction (PCR) was performed using Light cycler 480 SYBR GreenI master (Roche, 04707516001). Data were collected and analyzed on LightCycler480II (Roche). The housekeeping gene encoding GAPDH was used to normalize samples. Primers used including the followings: mus Atg7-F: GTTCGCCCCCTTTAATAGTGC, mus Atg7-R: TGAACTCCAACGTCAAGCGG; mus Gapdh-F: AGCTTGTCATCAACGGGAAG, mus Gapdh-R: TTTGATGTTAGTGGGGTCTCG; mus P15-F: TCTTGCATCTCCACCAGCTG, mus P15-R: CTCCAGGTTTCCCATTTAGC; mus P16-F: CGAACTCTTTCGGTCGTACCC, mus P16-R: CGAATCTGCACCGTAGTTGAGC; mus P19-F: GTTCTTGGTCACTGTGAGGATTCAG, mus P19-R: CCATCATCATCACCTGGTCCAG; mus P21-F: CCAGGCCAAGATGGTGTCTT, mus P21-R: TGAGAAAGGATCAGCCATTGC.

### Flow cytometry

Organs were digested by collagenase (Sigma, C2674) in 37 °C for about 1 hour to be disrupted into single cells. Flow cytometric cell sorting was performed using Beckman coulter (gallios). Hematopoietic cells were excluded by sorting with negative CD45 (Biolegend, 103106) and negative Ter119 (BD Biosciences, 563995). DCFH-DA (Thermo Fisher Scientific, D399) and Mitotracker deep red (Thermo Fisher Scientific, M22426) were stained for flow cytometric analysis according to the manufacturer’s instructions. γ-H2AX antibody was stained for flow cytometery after fixation and permeabilization by 4% PFA (Sinopharm chemical reagent, 80096628) and 0.1% saponin (Sigma, 47036). Apoptosis and necrosis were analyzed with flow cytometry by double staining of annexinV and PI (BD Biosciences, 556419) following manufacturer’s instructions. Thymus T cells and NK cells were analyzed with antibodies against in combinations of CD4 (BD Biosicences, 553049) and CD8 (Biolegend, 100706), or CD3 (eBioscience, 11-0032-82) and NK1.1 (Thermo Fisher Scientific, MM6628).

### Parabiosis

Parabiosis surgery followed previously described procedures (Kamran et al., 2013). Mirror-image incisions at the left and right flanks were made through the skin, and shorter incisions were made through the abdominal wall. The peritoneal openings of the adjacent parabionts were sutured together. Elbow and knee joints from each parabiont were sutured together. Each mouse was injected subcutaneously with Carprofen (Sigma) for pain and treated with sulfamethoxazole /trimethoprim oral suspension in their water bottle 2 mg sulfa/ml + 0.4 mg trim/ml for 10 days to prevent bacterial infections during recovery.

### Plasma collection and transplantation

Pooled mouse plasma was collected from Atg7^+/+^ or Atg7^-/-^ mice by intracardial bleed at time of euthanasia. Plasma was prepared from blood collected with sodium citrate followed by centrifugation at 2,000g, 4 °C. For plasma denaturation, plasma was heated for 2–3 min at 95 °C, followed by a short spin at 2,000g. All plasma aliquots were stored at −80 °C until use. Mice were systemically treated with plasma (100 μl per injection) isolated from mice by intravenously injections into the tail vein eight times over 24 days.

### Statistics

Results are shown as mean of the data from at least three independent experiments. For statistical comparison among groups, t test were used, with P <0.05 considered significant.
